# Exosomes miRNA-499a-5p targeted CD38 to alleviate anthraquinone induced cardiotoxicity: experimental research

**DOI:** 10.1097/JS9.0000000000001118

**Published:** 2024-01-26

**Authors:** Chunhua Ma, Zhaocong Yang, Jing Wang, Han She, Lei Tan, Qing Ye, Fei Wang, Xiaochun Feng, Xuming Mo, Kun Liu, Liangming Liu

**Affiliations:** a State Key Laboratory of Trauma, Burns and Combined Injury, Shock and Transfusion Research Department of Army Medical Center, Army Medical University, Chongqing; b Department of Cardiothoracic Surgery, Children’s Hospital of Nanjing Medical University, Nanjing; c School of Biology and Food Engineering, Institute of Pharmaceutical Pharmacology Research Center, Suzhou University, Suzhou, Anhui; d Department of Gynecology and Obstetrics; e Department of Cardiothoracic Surgery, Affiliated Hospital of Nantong University, Nantong, People’s Republic of China

**Keywords:** bone marrow mesenchymal stem cells, cardiotoxicity, doxorubicin, exosomes, miRNA-499a-5p

## Abstract

**Background:**

The purpose of this study was to investigate the effects of cardiac homing peptide (CHP) engineered bone marrow mesenchymal stem cells (BMMSc) derived exosomes (B-exo) loaded miRNA-499a-5p on doxorubicin (DOX) induced cardiotoxicity.

**Methods:**

miRNA chip analysis was used to analyze the differences between DOX induced H9c2 cells and control group. CHP engineering was performed on BMMSc derived exosomes to obtain C-B-exo. miRNA-499a-5p mimic was introduced into C-B-exo by electroporation technology to obtain C-B-exo-miRNA-499a-5p. DOX was used to establish a model of cardiotoxicity to evaluate the effects of C-B-exo- miRNA-499a-5p *in vivo* and *in vitro*. Western blot, immunohistochemistry, immunofluorescence, and other molecular biology methods were used to evaluate the role and mechanism of C-B-exo-miRNA-499a-5p on DOX induced cardiotoxicity.

**Results:**

miRNA chip analysis revealed that miRNA-499a-5p was one of the most differentially expressed miRNAs and significantly decreased in DOX induced H9c2 cells as compared to the control group. Exo-and B-exo have a double-layer membrane structure in the shape of a saucer. After engineering the CHP of B-exo, the results showed that the delivery of miRNA-499a-5p significantly increased and significantly reached the target organ (heart). The experimental results showed that C-B-exo-miRNA-499a-5p significantly improved electrocardiogram, decreased myocardial enzyme, serum and cardiac cytokines, improved cardiac pathological changes, inhibited CD38/MAPK/NF-κB signal pathway.

**Conclusions:**

In this study, C-B-exo-miRNA-499a-5p significantly improved DOX-induced cardiotoxicity via CD38/MAPK/NF-κB signal pathway, providing a new idea and method for the treatment of DOX induced cardiotoxicity.

## Introduction

Highlights

- miRNA499a-5p is a biomarker of cardiac toxicity caused by anthraquinone chemotherapeutic drugs.
- Cardiac homing peptide enhances exosome targeted delivery of miRNA499a-5p to cardiac tissue.
- Cardiac homing peptide-exo- miRNA499a-5p alleviated cardiac toxicity caused by anthraquinone compounds.

Doxorubicin (DOX), an anthracycline antibiotic found in mutant streptomyces, is one of the most effective chemotherapeutic drugs. It is commonly used to treat many solid tumors and malignant hematological diseases, including breast cancer, lung cancer, lymphoma, ovarian cancer, soft tissue sarcoma, and leukemia^1^. However, in clinical, it has been found that the use of DOX produced many toxic and side effects, such as hematopoietic suppression, nausea, vomiting, extravasation, and alopecia, and the most serious was cumulative and dose-dependent cardiotoxicity^2,3^. Clinically, the cardiotoxicity of DOX is diverse^4^. Although DOX has serious toxic and side effects, it is still one of the main drugs for treating cancer. However, its cardiotoxicity needs further elaboration.

Bone marrow mesenchymal stem cells (BMSCs) are mammalian cells, which can be differentiated into bone, muscle tissue, and connective tissue. At present, BMSCs, as a kind of stem cells with multidifferentiation function, are widely used in the treatment of cardiovascular diseases^5^. Exosomes are endogenous vesicular particles that have attracted attention in recent years. They exist in almost all biological fluids, including blood, urine, saliva, cerebrospinal fluid, and cell pretreatment media^6^. Exosomes from different cell sources can carry different bioactive substances, such as lipids, proteins, nucleic acids, etc., which act as mediators of information exchange between cells and participate in the regulation of various cell biological functions. Therefore, exosomes play an important role in many diseases^7^. The aim of this study was to investigate the effects of BMSCs derived exosomes carrying miRNA-499a-5p on DOX induced cardiotoxicity.

## Materials and methods

### Reagent

Creatine kinase MB (CK-MB) and lactate dehydrogenase (LDH) assay kits (Wuhan Huamei Bioengineering Co., Ltd.). Fetal bovine serum, phosphate buffer (PBS), and DMEM medium were purchased from America National Hyclone Company. Transfection reagent Transit-X2 was purchased from America Minas Company. Endotoxin-free plasmid small amount extraction kit was purchased from Qiagen Company of Germany. BCA protein quantitative kit was purchased from Beijing Pulilai Company. DOX was purchased from Sigma Company. Diamino polyethylene glycol was purchased from Yarebio Company. All antibodies were purchased from Cell Signaling Technology Company.

### Animals

C57BL/6J (male, 6 weeks, 16–18 g) were obtained from the SHANGHAI SLAC LABORATORY ANIMAL CO. LTD. WT mice (C57BL/6J, stock No: 000663, male, 6 weeks, 16–18 g) and CD38 KO mice (C57BL/6J, *CD38* ^*-/-*^ /J stock No: 020289, male, 6 weeks, 16–18 g) were obtained from the Cyagen Biosciences Inc. All animals’ operations were approved by the Research Council and Animal Care and Use Committee of our University (NT2023269). The work has been reported in accordance with the ARRIVE guidelines (Animals in Research: Reporting In Vivo Experiments)^8^.

### DOX stimulated H9c2 cells and differential miRNA identification

#### DOX stimulated H9c2 cells

H9c2 cells were cultured in DMEM medium containing 10% fetal bovine serum, 1% penicillin, and streptomycin in a 5% CO2 saturated humidity incubator at 37°C. The experiment was divided into two groups: control group and model (M) group (1 μmol/L DOX) for 24 h.

#### miRNA chip analysis

The miRNA-microarray expression profile chip of Shanghai Bohao biological company was used for the experiment. The brief process was as follows: the cells were labeled with fluorescence and then was subjected to chip hybridization and subjected to rolling hybridization at 55°C, 20 r/min, for 20 h in a rolling hybridization furnace. After hybridization was completed, the plates were washed in a washing tank. The chip results were scanned using Agilent Microarray Scanner and data were read using Feature Extraction software 10.7.1.1, and finally normalized using R language package and the algorithm used was Quantile. All miRNAs primer sequences are shown in Table 1.

**Table 1:** miRNAs primer sequence.

Gens	Primer sequence (5’-3’)
miRNA499a-5 F	5’-ATGTAGCGTGCGACCG-3'
miRNA499a-5 R	5’- CAGGCTGACGCACTCTGTGCT-3'
miRNA-21-5p F	5’-TAGCTTATCAGACTGATG-3’
miRNA-21-5p R	5’-CAGTGCGTGTCGTGGAGT-3’
miRNA-21 F	5’-ACACTCCAGCTGGGTAGCTTATCAGACTGA-3'
miRNA-21R	5’-CTCAACTGGTGTCGTGGAGTCGGCAATTCAGTTGAGTCAACATC-3'
miRNA-143 F	5’-ACATTGCAGCTGCCAAAAT-TT-3’
miRNA-143 R	5’-ACAGGCCAGCTTTGTA-3’
miRNA-147a F	5’-ATTGGCAAGATGTTCCTTGC-3’
miRNA-147a R	5’-GTGTGTGGAAATGCTTCTGC-3’
miRNA-146a F	5’-CACGGACCTGAAGAACACTGG-3’
miRNA-146a R	5’-AGAAATGAAATTAGAACACACATCAATCC-3’
miRNA-155 F	5’-ACCCTGCTGGATGAACGTAG-3’
miRNA-155 R	5’-CATGTGGGCTTGAAGTTGAG-3’
miRNA-494 F	5’-TTAATGCTAATCGTGACT-3’
miRNA-494 R	5’-ACCTGAGAGTAGACCAGA-3’
U6 F	5’-CCATCGGAAGCTCGTATACGAAATT-3’
U6 R	5’-GGCCTCTCGAACTTGCGTGTCAG-3’

Data can be requested through the corresponding author.

### Isolation and identification of BMSC and extraction of exosomes

#### Isolation and identification of BMSC

Mice were killed with isoflurane (50 mg/kg). After being soaked and disinfected with 75% ethanol for 5 min, bilateral femurs and tibias were aseptically taken out and brought back to the cell chamber. The muscle, fascia, and other connective tissues attached to the bone surface in the ultra-clean table were removed, the bone marrow cavity along the longitudinal axis of the long bond was cut, repeatedly. The bone marrow cavity was rinsed with 3 ml of low sugar DMEM complete medium containing 10% fetal bovine serum until the bone marrow cavity became white, the rinsing solution was sucked into a 50 ml plastic culture flask for use. The remaining bone tissue in the glass dish was cut into about 1 mm^3^ bone fragments, soaked in 0.1% type II collagenase, shaken and digested for 90 min. Finally, the digested bone fragments were inoculated into the culture flask with bone marrow liquid, and placed horizontally in the culture box for static culture. After 48 h, the first full amount of liquid shall be changed, and then half amount of liquid shall be changed once every other day.

#### Observation of cell morphology

The morphology, adherence, density, and fusion degree of primary and subculture cells were observed under a microscope.

#### Molecular detection of surface marker

The BMSCs were cultured to about 60% confluence, and then fluorescent CD90, CD73 and CD29, antibodies were added in turn. After incubation at 4°C for 40 min, CDs was added for flow cytometry detection.

#### Extraction of exosomes from BMSC

After 48 h of cell culture, the culture medium was collected and transferred to an ultrafiltration centrifuge tube. The culture medium was centrifuged at 4°C at 12736 r/min for 20 min to further remove cells and cell debris and then passed through 0.2 μM to remove particles >200 nm. Exosomes were collected by centrifugation at 34 288 r/min for 80 min at 4°C, and the resulting precipitates were exosomes (B-exo).

### Characterization of B-exo

#### Transmission electron microscope (TEM) examination

The B-exo was dropped on the wax tray, the surface with supporting film was contacted with the surface of the B-exo solution by the copper mesh, and it was taken out after standing for 3–5 min. The copper mesh was taken out, and the excess droplets were sucked out with filter paper strips and dried slightly. Three percent phosphotungstic acid solution was dropped on the wax dish. The copper mesh was adsorbed with the sample on the surface of the dye solution (B-exo) was in contact with the dye solution) for 3–5 min. The copper mesh was taken out, the excess droplets were sucked out with filter paper strips, and dried under incandescent lamps. TEM photographs were taken to observe the morphology of B-exo.

#### Nanoparticle tracking analysis (NTA)

The B-exo with 1×PBS buffer was diluted appropriately and particle size was analyzed (Zeta view system was calibrated with 110 nm polystyrene particles, and the temperature was maintained at 23 and 37°C).

#### Determination of exosomes surface markers

The expression of exosome marker proteins CD63, CD9, and TSG101 were detected by Western blot.

### B-exo loading with miRNA-499a-5p and engineered with CHP

#### B-exo loading with miRNA-499a-5p

miRNA-499a-5p mimics or miRNA-499a-5p negative control (NC) were transduced into exosomes by electro-transfer. The specific methods were as follows: the exosome suspension and electroporation buffer were mixed in the ratio of 1:1 for 5 min at room temperature, miRNA-499a-5p mimics or miRNA-499a-5p NC were added to the mixture (every 200 μl mixture contains 1 μmol miRNA-499a-5p mimics or 1 μmol miRNA-499a-5p NC). 200 μl the above mixed solution was added into the electric transfer tube, which was put into the gene pulser II electric transfer system at 150 V, 100 μF. RNase H was used to treat the exosome suspension after electro-transfer to remove the scattered miRNA-499a-5p NC and miRNA-499a-5p mimics that were not electro-transferred into exosomes. The exosomes of miRNA-499a-5p NC and miRNA-499a-5p mimics were named B-exo-NC and B-exo miRNA-499a-5p respectively. Detection of miRNA-499a-5p expression in exosomes by qPCR. miRNA-499a-5p primer sequence was shown in 5’-A T G T A G C G T G C G A C C G-3’, 5’-C A G G C T G A C G C A C T C T G T G C T-3’. U6: 5’- C C A T C G G A A G C T C G T A T A C G A A A T T -3’, 5’-G G C C T C T C G A A C T T G C G T G T C A G-3’.

#### CHP engineered B-exo-miRNA-499a-5p

1 μmol B-exo-miRNA-499a-5p was added to the CHP solution (1 μmol) and incubated at 4°C for 24 h. Then dialysis was performed to remove the unbound CHP, and freeze-drying was performed to obtain CHP engineered B-exo-miRNA-499a-5p named C-B-exo-miRNA-499a-5p.

### Localization of C-B-exo in vitro

#### DiR-labeled C-B-exo and B-exo

C-B-exo or B-exo was incubated with 1 mol fluorescent lipophilic tracer DiR at room temperature for 15 min and then DiR-C-B-exo and DiR-B-exo were obtained.

#### Small animal imaging analysis

60 μl of DiR-C-B-exo and 60 μl of DiR-B-exo were injected into the mice via tail vein respectively. After 2 h injection, the mice were put into a small animal living imager (IVIS SPECTRUM) for back and abdomen living imaging.

### Relationship between miRNA-499a-5p and DOX-induced cardiotoxicity

#### Search for miRNA-499a-5p target gene

The target gene of miRNA-499a-5p was calculated by using bioinformatics analysis method and miRbase and Targets-can software database.

#### Analysis of double luciferase reporter gene

The construction of plasmid was completed by Guangdong Ruibo biological Co., Ltd. Plasmid and miRNA-499a-5p were co-transfected as follows: 25 μl dilute miRNA-499a-5p NC or miRNA-499a-5p mimics with serum-free opti-MEM, and gently mixed with the gun head. 25 µl serum free opti-MEM was diluted with 1 µl reporter plasmid. Plasmids were divided into two types: wild-type (WT) and mutant plasmids (Mutant) were mixed. 25 μl serum free opti-MEM was diluted with 1.5 μl lipofectamine 3000. The final reaction system was 100 μl, and the remaining liquid was replaced by serum-free medium. 100 μl of fish phospholipase B was added to cells/each well, the suspension was added to 96 well plate (50 μl/per well), then 100 μl LAR-II was added, the fluorescein reaction intensity value was recorded at the same time, then 100 μl of reaction termination solution was added and finally the fluorescein reaction intensity value of fluorescein was recorded. The results were expressed by firefly fluorescein reaction intensity value/firefly fluorescein reaction intensity value.

#### Detection of target gene-related gene and protein expression by PCR and Western Blot

Total RNA of transfected cells was extracted by Trioal. After the concentration was determined, reverse transcription was performed by reverse transcription kit, and then fluorescent PCR was performed by AeCQ PCR SYBR Green Master Mix kit. The transfected cells were obtained, and the protein expression of related genes was detected by Western blot.

#### CD38 and MAPKP38 immunoprecipitation

An equal amount of protein lysate was added to the silicified EP tube, the specific antibody was then added (the control group was added with IgG with corresponding properties), and the protein-CD38/MAPKP38 beads were added for mixing overnight at 4°C. The sample was centrifuged at 4°C in the next day, beads with IP lysate was washed (if acetylation was detected, 1% deacetylase inhibitor was added), and the operation was repeated three times. Two×protein loading buffer was added, the sample was boiled in boiling water for denaturation, and immediately for Western blot.

### Establishment of DOX-induced cardiotoxicity models in vivo and in vitro

#### Establishment of DOX-induced cardiotoxicity mouse model

DOX-induced cardiotoxicity model of mice was established by intraperitoneal injection of DOX (6 mg/kg) at Day 1 and Day 4. The control mice (control group) were given the same amount of saline in the same way. Mice were divided into the following groups (*n*=10): Control mice (Ctr), DOX (M), DOX + B-exo-miRNA-499a-5p (20 µg/kg), DOX + C-B-exo-miRNA-499a-5p (20 µg/kg). WT mice, DOX+WT, DOX + CD38^-/-^, DOX + CD38^-/-^+ B-exo-miRNA-499a-5p (20 µg/kg), DOX + CD38^-/-^+ C-B-exo-miRNA-499a-5p (20 µg/kg). B-exo-miRNA-499a-5p and C-B-exo-miRNA-499a-5p (caudal vein injection) was given to mice for five consecutive days. The mice were euthanized with amobarbital sodium (30 mg/kg) and serum and hearts were taken for subsequent experiments.

#### *In vitro* model establishment

H9c2 cells were seeded at 6-well-plate at a density of 1×10^6^ cells/well for 24 h. After that, the cells were divided into following groups (*n*=10): Control (Ctr), DOX (M, 5 µM), DOX + B-exo-miRNA-499a-5p (30 μg/ml), DOX + C-B-exo-miRNA-499a-5p (30 μg/ml), DOX (M, 5 µM) + CD38 siRNA, DOX (M, 5 µM) + CD38 siRNA+ B-exo-miRNA-499a-5p (30 μg/mL), DOX (M, 5 µM) + CD38 siRNA+ C-B-exo-miRNA-499a-5p (30 μg/ml). The cells were treated with B-exo-miRNA-499a-5p (30 μg/ml) and C-B-exo-miRNA-499a-5p (30 μg/ml) for 24 h, and with 5 µM DOX were co-incubated for 24 h, while the control group was challenged with an equal PBS. Cell supernatant and cells were collected for subsequent detection.

### DOX induced long-term cardiac toxicity and intervention

The mice will be randomly divided into the following groups (*n*=10): control group, DOX group, DOX+B-exo-miRNA-499a-5p (20 µg/kg) group, DOX+C-B-exo-miRNA-499a-5p (20 µg/kg) group. Except for control group, mice were injected with DOX (6 mg/kg) intraperitoneally, once every 3 days, for a total of 10 times, 30 days in total. Meanwhile, B-exo-miRNA-499a-5p and C-B-exo-miRNA-499a-5p (caudal vein injection) was given to mice for 30 days. After 30 days, the mice were each day euthanized with amobarbital sodium (30 mg/kg) and serum and hearts were taken for subsequent experiments.

### Relevant index detection and relevant methods

#### Myocardial enzyme (CK), Phosphocreatine kinase isoenzyme and lactate dehydrogenase (LDH) test

The levels of CK, CK-MB, and LDH in serum were detected by related kits. The operation process strictly followed the instructions of the kits.

#### Cytokine measurement

The concentrations of IL-6, IL-1β, and TNF-α in serum, heart tissues and cell supernatant were analyzed by enzyme-linked immunosorbent assay (ELISA) kits according to the manufacturer’s instructions.

#### Hematoxylin-eosin (H&E) staining

The heart was fixed in 4% formalin solution, embedded in paraffin, cut into 4 μm thick sections, and placed on a glass slide for hematoxylin-eosin staining.

#### Echocardiographic determination

The mice in each group were examined by transthoracic color Doppler echocardiography. Left ventricular ejection fraction (LVEF), left ventricular fractional shortening (LVFS), left ventricular internal dimension-diastole (LVIDD), and left ventricular internal dimension-systole (LVIDS) were measured by ultrasound through the sternal left heart long axis section.

#### Cell viability

H9c2 cells were seeded at 96-well-plate at a density of 1×10^4^ cells/well for 24 h. After that, the cells were treated with B-exo-miRNA-499a-5p (30 μg/ml) and C-B-exo-miRNA-499a-5p (30 μg/ml) and with 5 µM DOX; were co-incubated for 24 h, while the control group was challenged with an equal PBS. Cell viability was detected by CCK-8 method.

#### Immunohistochemistry

CD38 protein in heart tissue was detected by immunohistochemistry, specific operation methods according to previous report^3^. Heart tissue blank slices were baked at 60°C for 1 h, then paraffin was removed by xylene, dehydrated by gradient ethanol and heated by sodium citrate buffer for antigen repair. After natural cooling to room temperature, it was cultured with 3% hydrogen peroxide for 10 min. Each section was sealed with 3% BSA at room temperature. After removing the blocking solution, the section was incubated with the primary antibody at 4°C overnight, the secondary antibody was incubated for 10 min, and PBS was washed three times, each time for 3 min, the third antibody was incubated for 10 min and washed with PBS for 3 min each time. Samples were stained with DAB and hematoxylin, dehydrated by gradient ethanol and xylene, dried, sealed with neutral resin, and the expression of related proteins in lung tissue was observed under light microscope.

#### Western blot analysis

CD38/MAPKP38/NF-κB signaling pathway related proteins in heart tissue and H9c2 cells were detected by Western blot analysis, specific operation methods according to previous report^3^. SDS-PAGE electrophoresis was performed on protein samples and transferred to PVDF membrane. The 5% skimmed milk powder was sealed at room temperature for 2 h, and incubated with related equal primary antibodies, respectively. The membrane was rinsed with TBST and then reacted with horseradish peroxidase coupled secondary antibody. The membrane was rinsed with TBST and then developed with enhanced chemiluminescence (ECL) luminescent reagent. The optical density of the main band was measured by gray-scale imaging software (UVP, UK) to calculate the expression level of the above proteins in lung tissue.

#### Immunofluorescence

The levels of CD38 in H9c2 cells were evaluated by immunofluorescence, specific operation methods according to previous report^3^. H9c2 cells were exposed to 5% Triton X-100 room temperature for 20 min, washed three times with PBS, and sealed at room temperature with sheep serum for 1 h and corresponding primary antibodies (all at 1:1000) were added and incubated overnight at 4°C. The next day, it was incubated at room temperature for 1 h, washed three times in PBS, labeled with fluorescent secondary antibody, incubated in dark at 37°C for 30 min, washed three times in PBS, and observed under a fluorescence microscope

### Statistical analysis

All data were expressed as mean±SD and analyzed by one-way analysis of variance (ANOVA) followed by Tukey multiple comparison test using GraphPad prism 8 (GraphPad Software, USA). A value of *P*<0.05 was considered statistically significant.

## Results

### Analysis of differential miRNAs between control H9c2 cells and DOX-induced H9c2 cells

**Figure 1:**
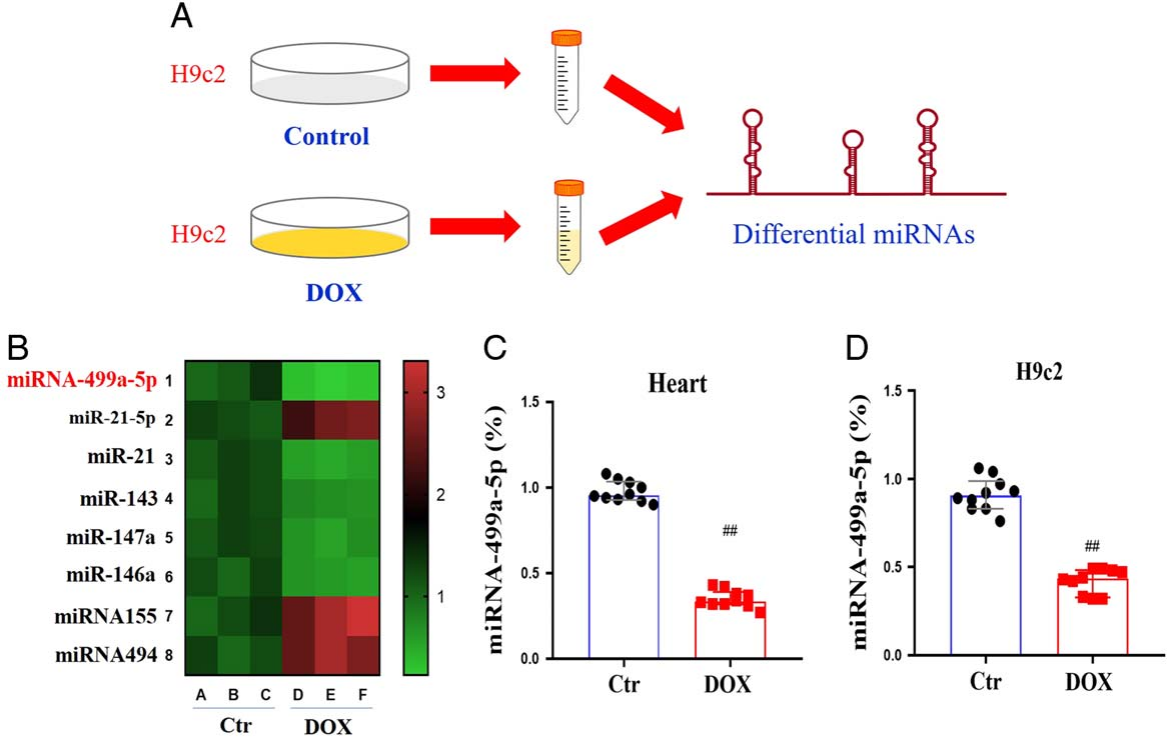
Differential miRNAs between control H9c2 cells and DOX-induced H9c2 cells (*n*=10). (A) Experimental process; (B) Differential miRNA hotspot map; (C-D) miRNA 499a-5p levels in mice and H9ce cells. All the data was presented as mean±SD. Compared with control group: ^#^ *P*<0.05, ^##^ *P*<0.01.

Since miRNA plays a role in regulating DOX-induced cardiotoxicity, this study used miRNA chip to detect the difference of differential miRNAs between control H9c2 cells and DOX-induced H9c2 cells (Fig. 1A). There were eight differentially expressed miRNAs between control H9c2 cells and DOX-induced H9c2 cells were detected. Among them, miRNA-499a-5p, miR-146a, miR-143 were significantly down-regulated. And miRNA-499a-5p was most down-regulated most obviously. The other five miRNAs were significantly up-regulated. Therefore, miRNA-499a-5p attracted our attention (Fig. 1B). In order to further confirm the down-regulation of miRNA-499a-5p expression in DOX-induced cardiotoxicity *in vivo* and *in vitro*, PCR was used to detect the expression of miRNA-499a-5p in DOX-induced cardiotoxicity *in vivo* and *in vitro*. The results showed that the expression of miRNA-499a-5p in DOX-induced models was significantly lower than that of control (Fig. 1C–D).

### Search for miRNA-499a-5p target gene

**Figure 2:**
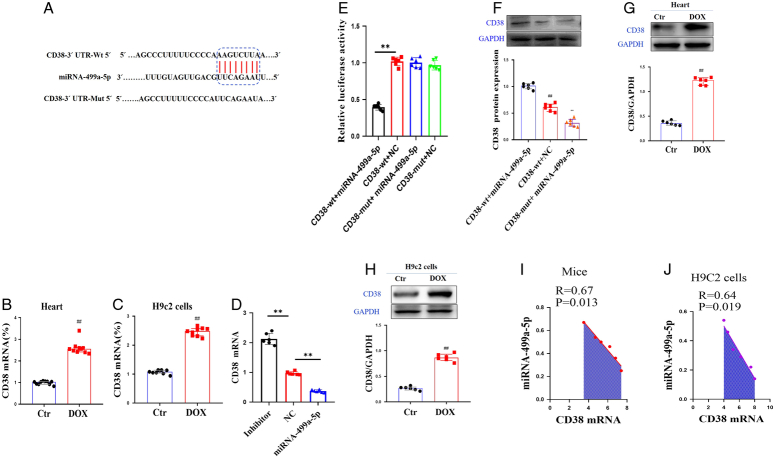
Search for miRNA-499a-5p target gene. (A)Bioinformatics analysis using miRBase, Targetscan, microRNA.org, starBase v2.0 softwares, A potential target of miRNA 499a-5p was found in CD38 mRNA; (B–C) CD38 miRNA levels in DOX-induced mice and DOX-induced H9c2 cells; (D–F) miRNA 499a-5p expression level under different conditions. All data were presented as mean±SD. Compared with *CD38*-wt+ miRNA 499a-5p group: ^*^ *P*<0.05, ^**^ *P*<0.01. (G–H) *CD38* protein expression in DOX-induced mice and DOX-induced H9c2 cells. All data were presented as mean±SD. Compared with Ctr: ^*^ *P*<0.05, ^**^ *P*<0.01. (I–J) Correlation between miRNA 499a-5p and CD38.

Bioinformatics analysis using miRbase and TargetScan software, a potential target of miRNA-499a-5p was found in CD38 mRNA (Fig. 2A).

In order to verify the changes of CD38, we investigated the expression of CD38 gene in DOX-induced cardiotoxicity *in vivo* and *in vitro*, and *in vitro* models, and found that in *in vivo* and *in vitro* M groups, CD38 mRNA significantly increased (Fig. 2B–C). Compared with the wild-type 3’-URT, the double luciferase activity of CD38-wt + miRNA-499a-5p group significantly decreased, and the double luciferase activity of mutant group did not significantly change (Fig. 2D–F).

The expression of CD38 protein was further detected, and the expression of CD38 proteins in DOX-induced cardiotoxicity *in vivo* and *in vitro* were investigated. The results showed that M groups *in vivo* and *in vitro*, CD38 protein was significantly increased (Fig. 2 G–H). These results suggested that the expression of miRNA-499a-5p was negatively correlated with the expression of CD38 (Fig. 2 I–J).

**Figure 3:**
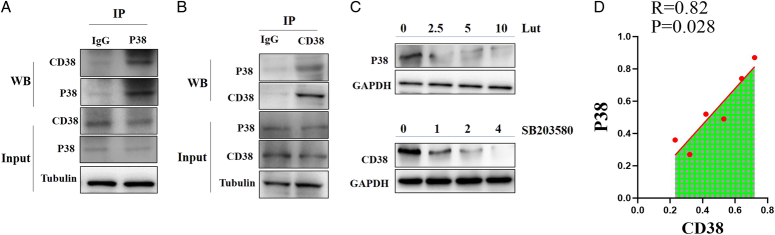
Immunoprecipitation of CD38 and P38. (A–B) The total protein cleaved by H9c2 cells was co precipitated with IgG, P38 antibody, or CD38 antibody of the control group, and then western blot was performed with P38 antibody or CD38 antibody, respectively, to confirm the binding of P38 to CD38. (C) CD38 inhibitor luteolinidin (Lut, 0, 2.5, 5, 10 µM) was used to treat H9c2 cells to detect the expression of P38, P38 inhibitor SB203580 was used to treat H9c2 cells to detect the expression of CD38. (D) Correlation between CD38 and P38.

Other literatures indicated that the expression of CD38 was MAPK pathway dependent. In LPS stimulated dendritic cells, p38 inhibitor effectively inhibited the upregulation of CD38 expression, ERK inhibitor also has a small inhibitory effect on CD38 expression, while JNK inhibitor has no obvious effect on CD38 expression^9^. Therefore, the interaction between CD38 and P38 protein was detected by immunoprecipitation. The result showed that CD and P38 could be endogenous bound into protein complexes (Fig. 3 A–B). These results suggest that there was an interaction between CD38 and P38.

To further confirm the relationship between CD38 and P38, in this experiment, CD38 inhibitor luteolinidin (Lut, 0, 2.5, 5, 10 µM) was used to treat H9c2 cells to detect the expression of P38. The results showed that Lut significantly inhibited the expression of P38 (Fig. 3 C). Conversely, in this experiment, P38 inhibitor SB203580 (0, 1, 2, 4 µM) was used to treat H9c2 cells to detect the expression of CD38, the results showed that SB203580 significantly inhibited the expression of CD38 in a dose-dependent manner (Fig. 3 C). The above results showed that CD38 was positively correlated with p38 and there was an interaction between CD38 and P38 (Fig. 3 D).

### Characterization of B-exo and BMSC

#### TME observation of B-exo

**Figure 4:**
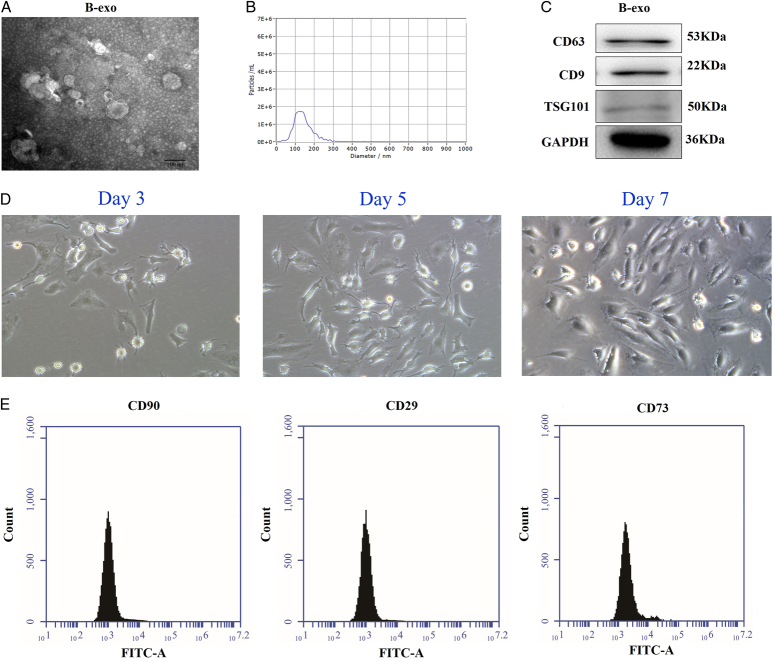
Characterization of B-exo and BMSC. (A–B) TEM of B-exo; (B) B-exo particle size analysis; (C) B-exo marker proteins; (D) Morphology of BMSC in different days under light microscope; (E) BMMCs marker.

TEM results clearly show that the BMSC derived exosomes are in the shape of saucers, with double-layer membrane structure and complete membrane structure (Fig. 4A).

#### Nanoparticle tracking analysis of B-exo

Through NTA particle size analysis, the B-exo with an average particle size of 79.6 nm (Fig. 4B). The particle size distribution conforms to the exosome particle size.

#### B-exo marker protein test results

As shown in Figure 4C, the expressions of marker proteins of B-exo were detected by Western blot. The results showed that CD63, CD9, and TSG101 were highly expressed, which was consistent with the characteristics of exosome surface markers.

#### Identification of BMSC

As shown in Figure 4D, under light microscope, it was found that BMMCs gradually generated spindle cells from day 3 to day 7. The results of flow cytometry showed that, CD29, CD90, and CD73 were positive (Fig. 4E).

### C-B-exo-miRNA-499a-5p can reach the target organ-heart tissue of mice and cardiac release of miRNA-499a-5p

**Figure 5:**
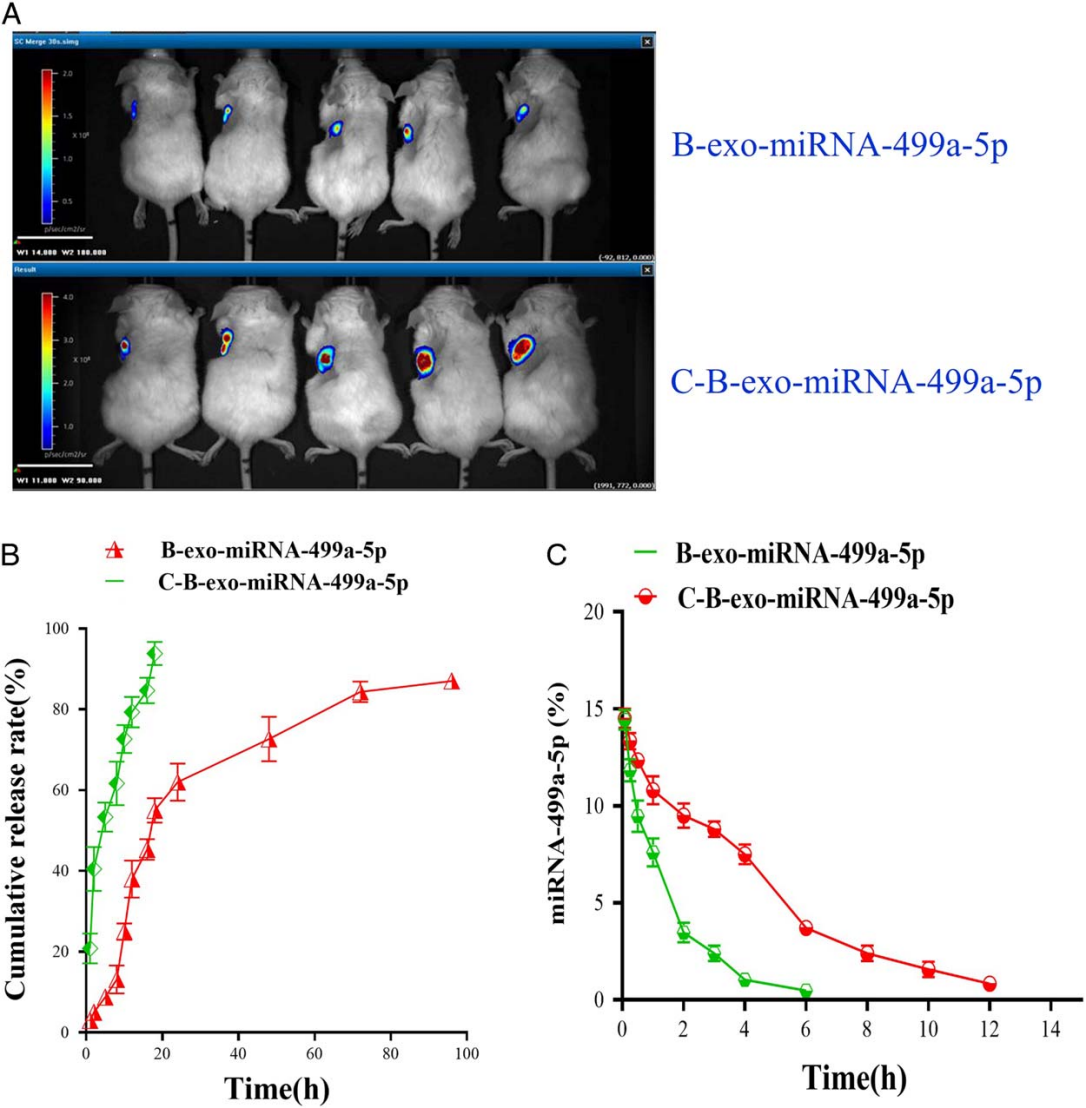
B-exo-miRNA-499a-5p and C-B-exo-miRNA-499a-5p target heart in vivo and release in vivo. (A)B-exo-miRNA-499a-5p cardiac targeting; (B) C-B-exo-miRNA-499a-5p cardiac targeting; (C) miRNA-499a-5p cumulative release rate; (D) In vivo metabolic curve of miRNA-499a-5p.

DiR-C-B-exo were injected into the tail vein of the mice. After in vivo imaging, it was found that fluorescence showed in the heart of mice. The results showed that C-B-exo could reach the target organ-heart tissue (Fig. 5A). It was suggested that CHP contributed to the targeted delivery of miRNA-499a-5p to the heart via B-exo.

As shown in Figure 5B-C, the levels of miRNA-499a-5p were significantly rapidly increased in heart of C-B-exo-miRNA-499a-5p group, and the decrease rate of miRNA-499a-5p content was very slow in heart of C-B-exo-miRNA-499a-5p group. While, the levels of miRNA-499a-5p were increased slowly in in heart of B-exo-miRNA-499a-5p group and the decrease rate of miRNA-499a-5p content was fast heart of B-exo-miRNA-499a-5p group.

### The effects of C-B-miRNA-499a-5p on DOX-induced cardiotoxicity *in vivo and in vitro*

#### The effects of C-B-miRNA-499a-5p on CK, CK-MB, and LDH in DOX-induced mice

**Figure 6:**
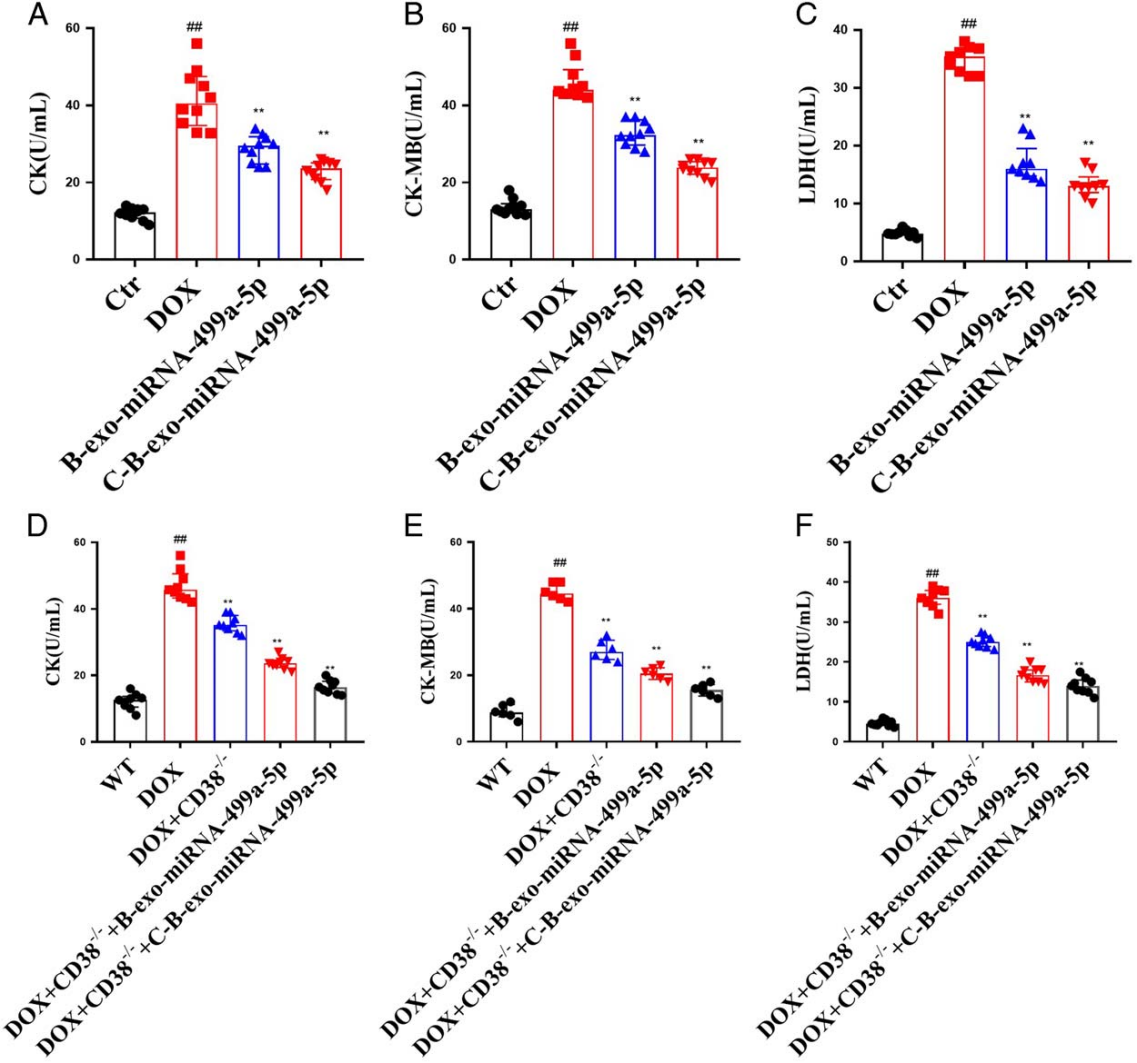
The effects of C-B-exo-miRNA-499a-5p on CK, CK-MB, LDH (*n*=10). (A–C) CK, CK-MB, LDH in DOX-induced mice. (D–F) CK, CK-MB, LDH in DOX-induced CD38^-/-^ mice. All data were presented as mean±SD. Compared with control group: ^#^ *P*<0.05, ^##^ *P*<0.01. Compared with model group: ^*^ *P*<0.05, ^**^ *P*<0.01.

As shown in Figure 6 A–F, the levels of CK, CK-MB, and LDH in DOX-induced mice were markedly increased in DOX group than control group. Meanwhile, B-exo-miRNA-499a-5p and C-B-exo-miRNA-499a-5p markedly reduced the levels of CK, CK-MB, and LDH in DOX-induced mice. And the effect of C-B-exo-miRNA-499a-5p was better than that of B-exo-miRNA-499a-5p, suggesting that CHP was helpful for targeted delivery of miRNA-499a-5p.

#### The effects of C-B-miRNA-499a-5p on heart histopathology and echocardiography in DOX-induced mice

The cardiomyocytes in the control group were evenly arranged, with normal morphology and no abnormal morphological cells. In the M group, myocardial cell edema, vacuolar degeneration, disordered arrangement, cell proliferation, and even interstitial fibrosis appeared. In B-exo-miRNA-499a-5p and C-B-exo-miRNA-499a-5p groups mice, cardiomyocytes were arranged evenly, edema was reduced, vacuolar degeneration was reduced, and cell proliferation was not obvious.

**Figure 7:**
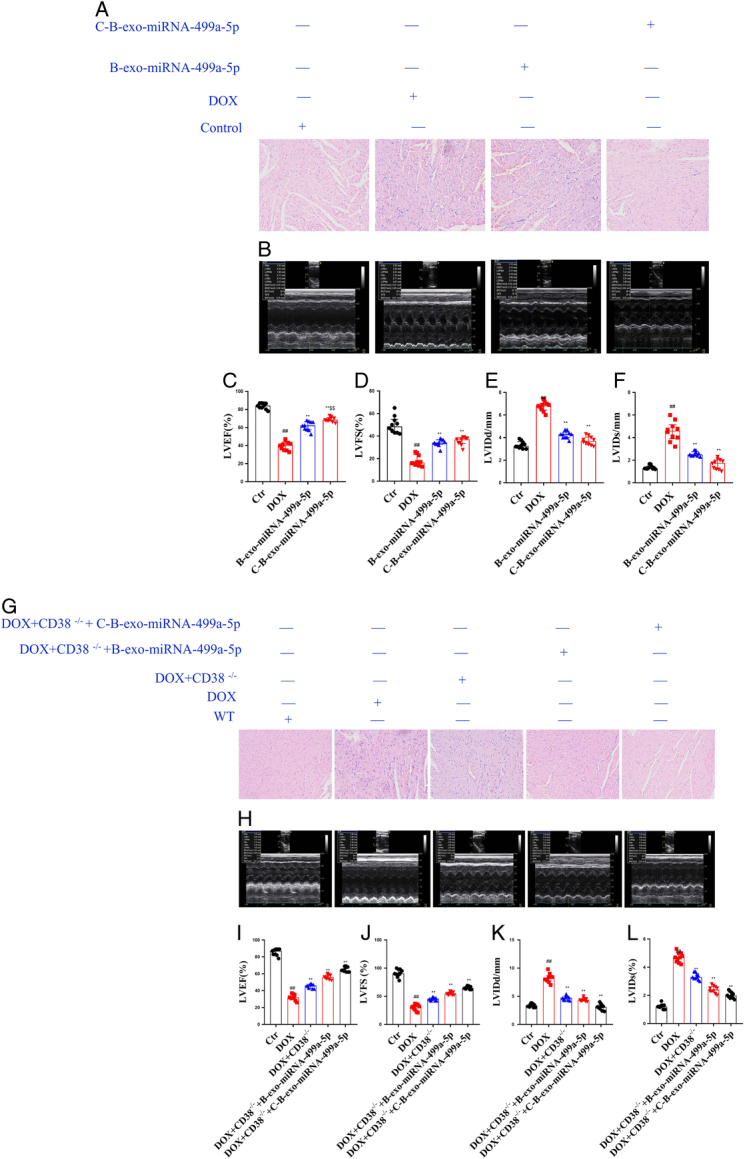
The effects of C-B-exo-miRNA-499a-5p on heart histopathology Echocardiography (*n*=10). (A) Representative heart tissue sections photomicrographs for hematoxylin-eosin (H&E) staining in DOX-induced mice (B–F) echocardiography in DOX-induced mice; (G) Representative heart tissue sections photomicrographs for hematoxylin-eosin (H&E) staining in DOX-induced CD38^-/-^ mice; (H–L) echocardiography in DOX-induced CD38^-/-^ mice.

Echocardiography is a common method to evaluate cardiac function. Compared with the control group, LVEF and LVFS of mice in DOX (M) group were significantly decreased, and LVIDS were significantly increased. Compared with DOX group, LVEF and LVFS were significantly increased and LVIDS were significantly decreased in B-exo-miRNA-499a-5p and C-B-exo-miRNA-499a-5p groups (Fig. 7).

#### The effects of C-B-miRNA-499a-5p on cytokine in DOX mice

**Figure 8:**
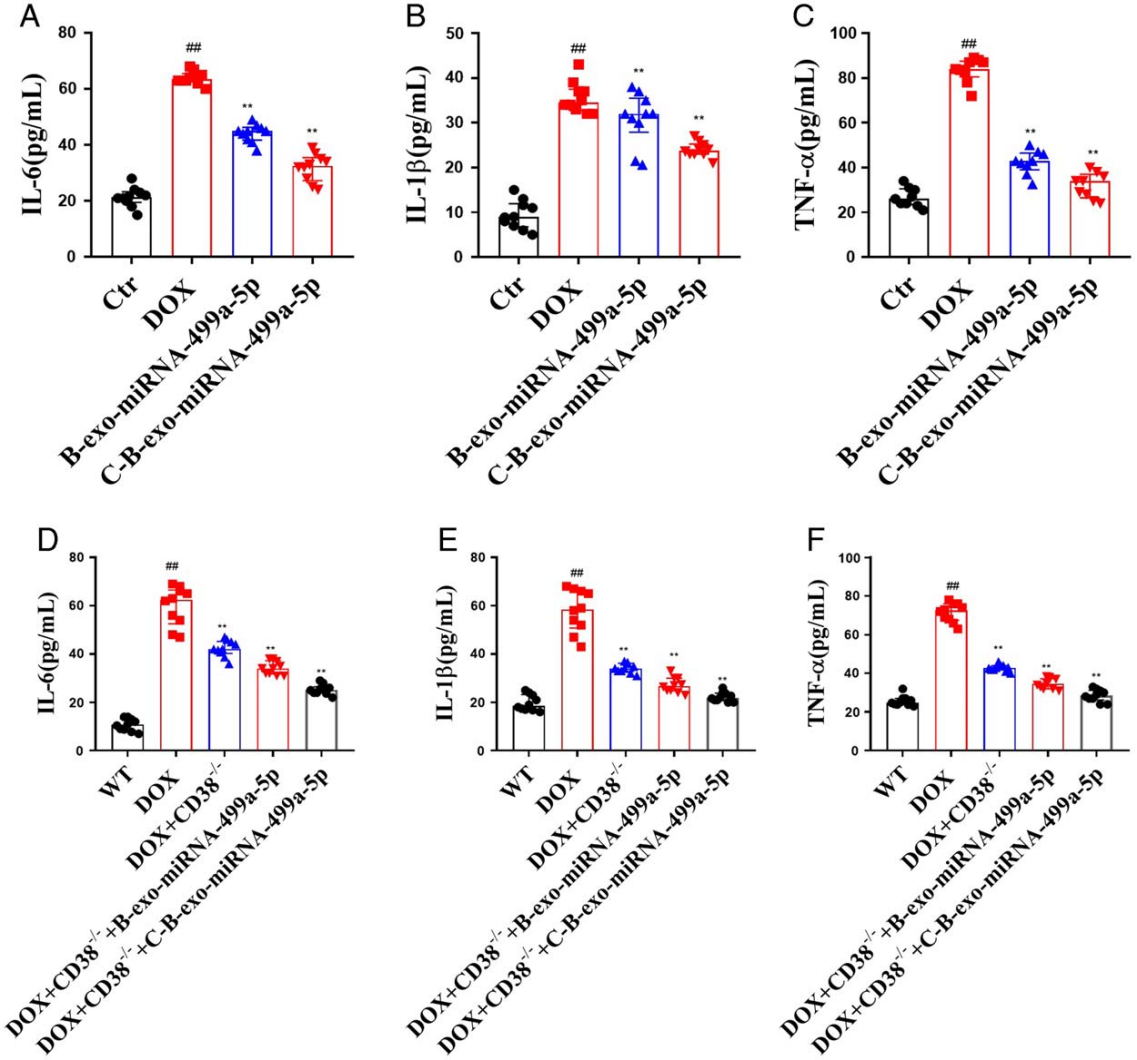
The effects of C-B-miRNA-499a-5p on cytokine in DOX mice (*n*=10). (A–C) The levels of TNF-α, IL-1β, and IL-6 in serum of DOX-induced mice; (D–F) The levels of TNF-α, IL-1β, and IL-6 in serum of DOX-induced CD38^-/-^ mice. All data were presented as mean±SD. Compared with control group: ^#^ *P*<0.05, ^##^ *P*<0.01. Compared with model group: ^*^ *P*<0.05, ^**^ *P*<0.01.

In order to evaluate inflammatory reaction, cytokines (TNF-α, IL-1β, and IL-6) were detected. As expected, the levels inflammatory cytokines TNF-α, IL-1β, and IL-6 were increased in serum in DOX (M) mice compared with control group. Compared with DOX (M) group, B-exo-miRNA-499a-5p and C-B-exo-miRNA-499a-5p substantially decreased the levels of TNF-α, IL-1β, IL-6 in serum (Fig.8 A–F).

#### The effects of C-B-miRNA-499a-5p on CD38/MAPKP38/NF-κB pathway in DOX mice

**Figure 9:**
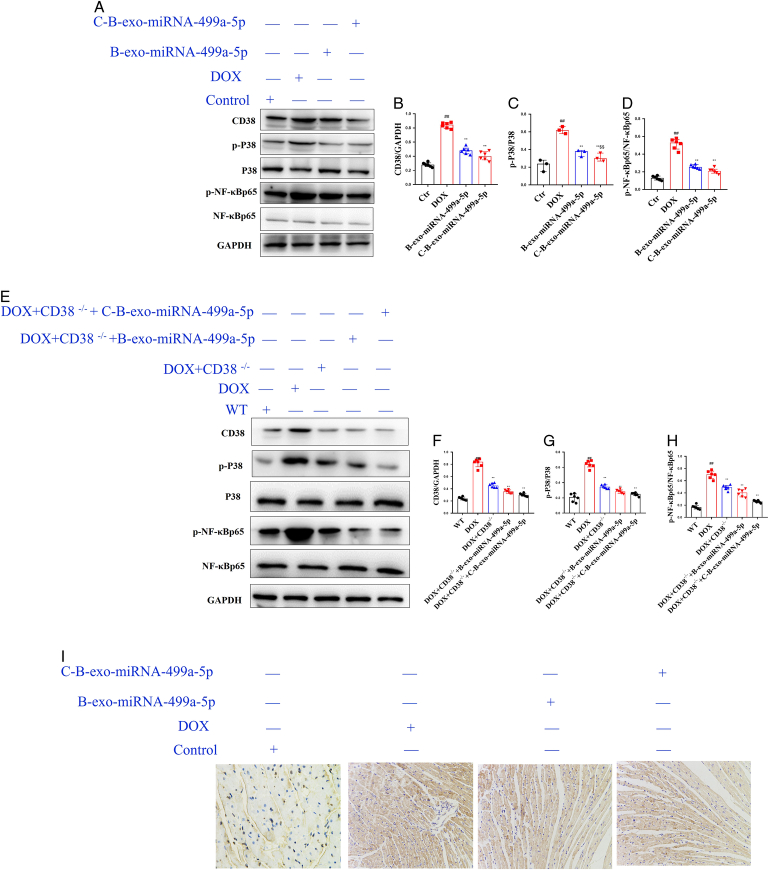
The effects of C-B-miRNA-499a-5p on CD38/MAPKP38/NF-κB pathway in DOX-induced mice (*n*=10). (A–D) The levels of CD38, p-P38, and p-NF-κBP38 in heart of DOX-induced mice; (E–H) The levels of CD38, p-P38, and p-NF-κBP38 in heart of DOX-induced CD38^-/-^ mice; (I) Immunohistochemistry of CD38 in lung (*n*=3). Original magnification: 200, scale bar: 20 μm. All data were presented as mean±SD. Compared with control group: ^#^ *P*<0.05, ^##^ *P*<0.01. Compared with model group: ^*^ *P*<0.05, ^**^ *P*<0.01.

As shown in Figure 9 A–H, compared with control group, the levels of CD38, p-P38, and p-NF-κBp65 were increased in DOX (M) group. Compared with DOX (M) group, B-exo-miRNA-499a-5p and C-B-exo-miRNA-499a-5p substantially decreased the levels of CD38, p-P38, and p-NF-κBp65. In immunohistochemistry experiments (Fig. 9 I), as expected, B-exo-miRNA-499a-5p and C-B-exo-miRNA-499a-5p significantly decreased the level of CD38 in DOX mice.

#### The effects of C-B-miRNA-499a-5p on cell viability and cytokine in DOX-induced H9c2 cells

**Figure 10:**
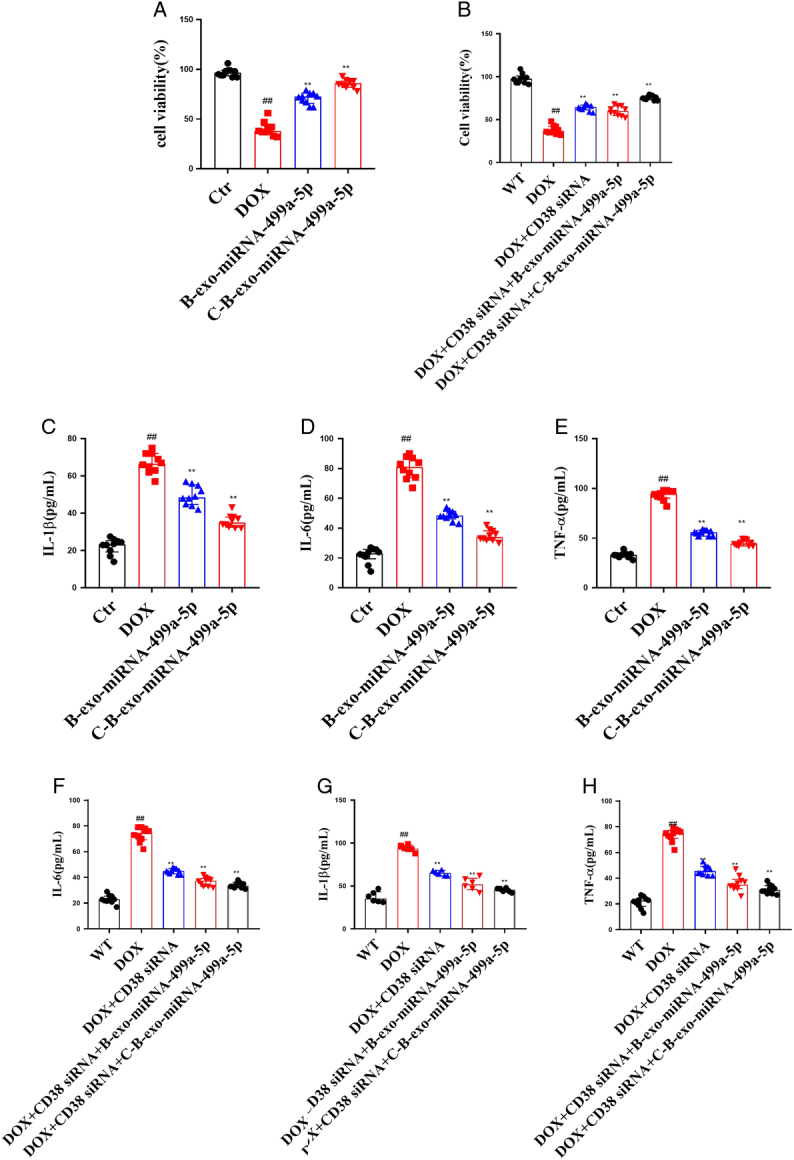
The effects of C-B-miRNA-499a-5p on cell viability and cytokine in DOX-induced H9c2 cells (*n*=10). (A-B) Cell viability; (C–E) The levels of TNF-α, IL-1β, and IL-6 in DOX-induced H9c2 cells; (F–H) The levels of TNF-α, IL-1β, and IL-6 in DOX-induced CD38 siRNA H9c2 cells. All data were presented as mean±SD. Compared with control group: ^#^ *P*<0.05, ^##^ *P*<0.01. Compared with model group: ^*^ *P*<0.05, ^**^ *P*<0.01.

As shown in Figure 10, DOX significantly increased levels of TNF-α, IL-1β, IL-6 in cell supernatant of H9c2 cells and the decrease of cell viability. Compared with DOX group, B-exo-miRNA-499a-5p and C-B-exo-miRNA-499a-5p significantly decreased the levels of TNF-α, IL-1β, and IL-6 and increased cell viability.

#### The effects of C-B-miRNA-499a-5p on CD38/MAPKP38/NF-κB pathway in DOX-induced H9c2 cells

**Figure 11:**
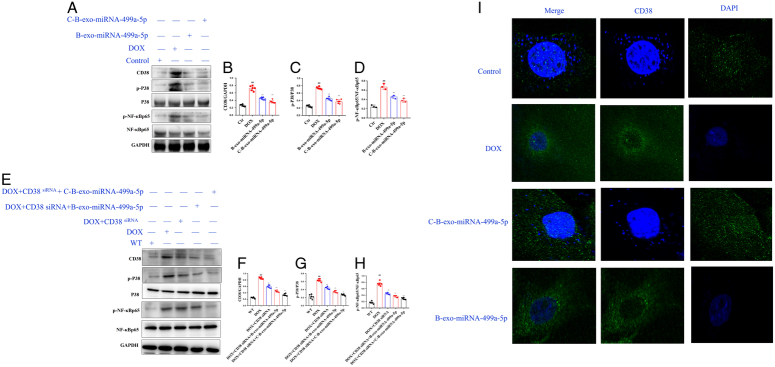
The effects of C-B-miRNA-499a-5p on CD38/MAPKP38/NF-κB pathway in DOX-induced H9c2 cells (*n*=10). (A–D) The levels of CD38, p-P38, and p-NF-κBP38 in heart of DOX-induced H9c2 cells; (E–H) The levels of CD38, p-P38, and p-NF-κBP38 in heart of DOX-induced CD38 siRNA H9c2 cells; (H) Immunofluorescence of CD38 in heart (*n*=3). Original magnification: 200, scale bar: 20 μm. All data were presented as mean±SD. Compared with control group: ^#^ *P*<0.05, ^##^ *P*<0.01. Compared with model group: ^*^ *P*<0.05, ^**^ *P*<0.01.

As shown in Figure 11A–H, compared with control group, the levels of CD38, p-P38, and p-NF-κBp65 were significantly increased in DOX group. Compared with DOX group, B-exo-miRNA-499a-5p and C-B-exo-miRNA-499a-5p significantly decreased the levels of CD38, p-P38, and p-NF-κBp65. In immunofluorescence experiments (Fig. 11 I), as expected, B-exo-miRNA-499a-5p and C-B-exo-miRNA-499a-5p significantly decreased the level of CD38 in DOX-induced H9c2 cells.

### The effects of C-B-miRNA-499a-5p on DOX induced long-term cardiac toxicity

#### CK, CK-MB, and LDH in DOX induced long-term cardiac toxicity mice

**Figure 12:**
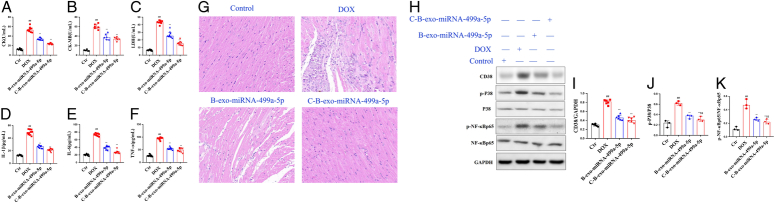
The effects of C-B-miRNA-499a-5p on DOX induced long-term cardiac toxicity. (A–C) CK, CK-MB, and LDH; (D-F) The levels of TNF-α, IL-1β, and IL-6; (G) Representative heart tissue sections photomicrographs for hematoxylin-eosin (H&E) staining; (H–K) The levels of CD38, p-P38, and p-NF-κBP38 in heart. All data were presented as mean±SD. Compared with control group: ^#^ *P*<0.05, ^##^ *P*<0.01. Compared with model group: ^*^ *P*<0.05, ^**^ *P*<0.01.

As shown in Figure 12 A–C, the levels of CK, CK-MB, and LDH in DOX-induced mice were significantly increased than control group. B-exo-miRNA-499a-5p and C-B-exo-miRNA-499a-5p significantly reduced the levels of CK, CK-MB, and LDH.

Cytokine: Compared with control group, the levels of cytokines TNF-α, IL-1β, and IL-6 were significantly increased in serum in DOX (M) mice. Compared with DOX (M) group, B-exo-miRNA-499a-5p and C-B-exo-miRNA-499a-5p significantly decreased the levels of TNF-α, IL-1β, and IL-6 in serum (Fig.12 D–F).

Heart histopathology: The cardiomyocytes in the control group were evenly arranged, with normal morphology and no abnormal morphological cells. In the M group, myocardial cell edema, vacuolar degeneration, disordered arrangement, cell proliferation, and even interstitial fibrosis appeared. In B-exo-miRNA-499a-5p and C-B-exo-miRNA-499a-5p groups mice, cardiomyocytes were arranged evenly, edema was reduced, vacuolar degeneration was reduced, and cell proliferation was not obvious (Fig. 12H-K).

CD38/MAPKP38/NF-κB pathway: Compared with control group, the levels of CD38, p-P38, and p-NF-κBp65 were significantly increased in DOX (M) group. Compared with DOX (M) group, B-exo-miRNA-499a-5p and C-B-exo-miRNA-499a-5p significantly decreased the levels of CD38, p-P38, and p-NF-κBp65 (Fig. 12H-K).

## Discussion

DOX is a widely used anticancer drug. However, DOX induced cardiomyopathy is a major obstacle to its use in cancer chemotherapy. Although many mechanisms have been demonstrated to be related to DOX induced cardiotoxicity, the exact and complete mechanism is unclear^10^, and the current treatment is not very satisfactory. In this study, a new therapeutic method was proposed, which is engineered exosomes loaded miRNA to the heart to treat DOX induced cardiotoxicity.

MicroRNAs (microRNAs, miRNAs) are a class of endogenous small molecule non coding RNAs with a length of about 20–25 nucleotides, which mediate and regulate a variety of pathophysiological processes in vivo^11,12^. In this study, the DOX induced H9c2 cell injury model was established and eight different miRNAs were identified by miRNA chip technology of which miRNA-499a-5p was the most different miRNA. It was further found that the expression of miRNA-499a-5p was significantly decreased in the DOX induced cardiotoxicity model. It was suggested that miRNA-499a-5p may be a potential target for intervention of DOX induced cardiotoxicity.

Extracellular vesicles are receiving increasing attention in heart related diseases. The cardioprotective effect of exosomes originally came from stem cell transplantation after myocardial infarction^13,14^ and exosomes are nano vesicles secreted by cells. It has been proved that exosomes are a natural carrier for drug delivery^15,16^. In addition, exosomes have high affinity and natural targeting ability. For example, exosomes can cross the blood-brain barrier and be selectively taken up by glial cells^17^. Exosomes have the intrinsic ability to carry and protect miRNA, cross biological barriers, and transport miRNA to rake cells for a long distance, and are nonimmunogenic to the host, exosomes can be used as a new delivery vehicle for the therapeutic use of miRNA. In this study, miRNA-499a-5p mimic was electro-transferred to exosomes. In addition, in order to deliver miRNA-499a-5p to the heart, the exosomes were engineered with the cardiac homing peptide to deliver miRNA-499a-5p to the heart.

In this study, we found that the target gene of miRNA-499a-5p was CD38 via bioinformatics. Previous studies have shown that miRNA-499a-5p was differentially expressed in cardiomyocytes and is closely related to a variety of heart diseases^17^. It has been reported that miRNA-499a-5p reduced cardiomyocyte apoptosis and lactate dehydrogenase (LDH) activity induced by hypoxia/reoxygenation by downregulating CD38 protein, thereby alleviating cardiomyocyte injury^18^. This study found that miRNA-499a-5p was negatively correlated with CD38 and MAPKp38 was positively correlated with CD38 expression. B-exo-miRNA-499a-5p and C-B-exo-miRNA-499a-5p significantly inhibited CD38/MAPK/NF-κB pathway to alleviate DOX-induced cardiotoxicity.

## Conclusions

In conclusion, this study found that miRNA-499a-5p is a potential marker of DOX induced cardiotoxicity, and the targeted delivery of miRNA-499a-5p via engineered exosomes effectively improved DOX induced cardiotoxicity. This study provides a new treatment for DOX induced cardiotoxicity.

### Limitations and prospect

Limitations: (1) The discovery of miRNA-499a-5p is based on the establishment of a DOX induced H9c2 cell model *in vitro*. Next, we will conduct validation of miRNA-499a-5p for clinical DOX induced cardiotoxicity patients. (2) This study only focused on the role of miRNA-499a-5p, and did not investigate other changed miRNAs, which has certain limitations. Therefore, in the future, we will conduct in-depth research on other miRNAs in DOX induced cardiotoxicity.

Prospects: (1) The discovered miRNA-499a-5p in this study can serve as a potential clinical diagnostic criterion. In clinical practice, a decrease in miRNA-499a-5p was found after DOX chemotherapy, which can provide a warning for DOX induced cardiac toxicity. (2) This study provides a rapid detection method for DOX induced cardiac toxicity in clinical practice. In clinical practice, DOX induced cardiac toxicity can be quickly warned.

## Ethical approval

All animals’ operations were approved by the Research Council and Animal Care and Use Committee of Nantong University (NT2023269).

## Sources of funding

None.

## Author contribution

C.M., Z.Y., and J.W.: did the experiments; H.S., L.T., Q.Y., F.W., and X.F.: collected data and data analysis; X.M., K.L., L.L.: designed experiments and wrote the paper.

## Conflicts of interests disclosure

All authors have no conflict of interest.

## Research registration unique identifying number (UIN)

1. Name of the registry: not applicable.
2. Unique identifying number or registration ID: not applicable.
3. Hyperlink to your specific registration (must be publicly accessible and will be checked): not applicable.

## Guarantor

Chunhua Ma.

## Availability of data and materials

Availability of data and materials can be obtained from corresponding author.

## References

[1] Renu K V.G A P.B T. Molecular mechanism of doxorubicin-induced cardiomyopathy-an update. Eur J Pharmacol 2018;818:241–253. 29074412 10.1016/j.ejphar.2017.10.043

[2] Nebigil CG D´esaubry L. , Updates in anthracycline-mediated cardiotoxicity. Front Pharmacol 2018;9:1262. 30483123 10.3389/fphar.2018.01262 PMC6240592

[3] Zhao N Li Q Sui H. Role of oxidation-dependent CaMKII activation in the genesis of abnormal action potentials in atrial cardiomyocytes: a simulation study. BioMed Res Int 2020;2020:1597012. 32685443 10.1155/2020/1597012 PMC7327560

[4] Henriksen PA. Anthracycline cardiotoxicity: an update on mechanisms, monitoring and prevention. Heart 2018;104:971–977. 29217634 10.1136/heartjnl-2017-312103

[5] Csapo M Lazar L. Chemotherapy-induced cardiotoxicity: pathophysiology and prevention. Clujul Med 2014;87:135–142. 26528012 10.15386/cjmed-339 PMC4508592

[6] Curigliano G Cardinale D Dent S. Cardiotoxicity of anticancer treatments: epidemiology, detection, and management. CA Cancer J Clin 2016;66:309–325. 26919165 10.3322/caac.21341

[7] Gianni L Herman EH Lipshultz SE. Anthracycline cardiotoxicity: from bench to bedside. J Clin Oncol 2008;26:3777–3784. 18669466 10.1200/JCO.2007.14.9401 PMC3018290

[8] Kilkenny C Browne WJ Cuthill IC. Improving bioscience research reporting: the ARRIVE Guidelines for reporting animal research. PLoS Biol 2010;8:e1000412. 20613859 10.1371/journal.pbio.1000412 PMC2893951

[9] Salvatorelli E Menna P Chello M. , Modeling human myocardium exposure to doxorubicin defines the risk of heart failure from lowdose doxorubicin. J Pharmacol Exp Ther 2017;362:263–270. 28559479 10.1124/jpet.117.242388

[10] An J Sheikh MS. Toxicology of trastuzumab: an insight into mechanisms of cardiotoxicity. Curr Cancer Drug Targets 2019;19:400–407. 29189161 10.2174/1568009618666171129222159

[11] Yang Y Bu P. Progress on the cardiotoxicity of sunitinib: prognostic significance, mechanism and protective therapies. Chem Biol Interact 2016;257:125–131. 27531228 10.1016/j.cbi.2016.08.006

[12] Yuan C Parekh H Allegra C. 5-FU induced cardiotoxicity: case series and review of the literature. Cardiooncology 2019;5:13. 32154019 10.1186/s40959-019-0048-3 PMC7048125

[13] Chustecka Z. Cardiotoxicity of imatinib is a” surprise. Nat Med 2011;35:36–37.

[14] Hawkes EA Okines AF Plummer C. Cardiotoxicity in patients treated with bevacizumab is potentially reversible. J Clin Oncol 2011;29:e560–e562. 21606423 10.1200/JCO.2011.35.5008

[15] Martínez-Mateo V Anguita M Del Campo L. Case report of cisplatin-induced cardiotoxicity: a side effect to monitor closely. J Heart Health 2017;3.

[16] Kelleni MT Abdelbasset M. Drug induced cardiotoxicity: mechanism, prevention and management, IntechOpen, London, UK, 2018

[17] Ayza MA Zewdie KA Tesfaye BA. The role of antioxidants in ameliorating cyclophosphamide-induced cardiotoxicity. Oxid Med Cell Longev 2015;2020:4965171. 10.1155/2020/4965171 PMC7238386 32454939

[18] Pizzino F Vizzari G Bomzer CA. Diagnosis of chemotherapy-induced cardiotoxicity. J Patient Cent Res Rev 2014;1:121–127.

